# Does chronic kidney disease affect the short-term outcomes and prognosis of colorectal cancer surgery? A propensity score matching analysis

**DOI:** 10.3389/fonc.2024.1400313

**Published:** 2024-07-03

**Authors:** Shu-Pei Qu, Si-Qi Rao, Zhan-Xiang Hai, Chun-Yi Wang

**Affiliations:** Department of Gastrointestinal Surgery, The First Affiliated Hospital of Chongqing Medical University, Chongqing, China

**Keywords:** chronic kidney disease, colorectal cancer, surgery, prognosis, propensity score matching

## Abstract

**Purpose:**

The aim of this study was to analyze the effect of chronic kidney disease (CKD) on the short-term outcomes and prognosis of colorectal cancer (CRC) patients who underwent primary surgery.

**Methods:**

CRC patients who underwent radical surgery were included from Jan 2011 to Jan 2020 in a single hospital. The short-term outcomes and prognosis were compared between the CKD group and the Non-CKD group using propensity score matching (PSM) analysis.

**Results:**

A total of 4056 patients undergoing CRC surgery were included, including 723 patients in the CKD group and 3333 patients in the Non-CKD group. After 1:1 PSM, there were 666 patients in each group, respectively. No significant difference was found in baseline characteristics between the two groups. (p>0.05). After PSM, the CKD group had a longer postoperative hospital stay (P=0.009) and a higher incidence of overall complications (p=0.050). Cox analysis was performed on matched patients to find predictors of overall survival (OS) and disease-free survival (DFS). We found that age (p<0.01, HR=1.045, 95% CI=1.028–1.062), tumor stage (p<0.01, HR=1.931, 95% CI=1.564–2.385) and overall complications (p<0.01, HR=1.858, 95% CI=1.423–2.425) were independent predictors of OS. Age (p<0.01, HR=1.034, 95% CI=1.020–1.049), tumor stage (p<0.01, HR=1.852, 95% CI=1.537–2.231), and overall complications (p<0.01, HR=1.651, 95% CI=1.295–2.10) were independent predictors of DFS. However, CKD was not an independent predictor of OS or DFS (OS: p=0.619, HR=1.070, 95% CI=0.820–1.396; DFS: p=0.472, HR=1.092, 95% CI=0.859–1.389).

**Conclusion:**

CKD prolonged postoperative hospital stay; however, CKD might not affect major postoperative complications, OS or DFS of CRC.

## Introduction

According to the global statistics released by the International Agency for Research on Cancer in 2022, the new cases of colorectal cancer (CRC) accounted for 9.6% of the new cases of malignant tumors in the world annually, ranking second only to breast and lung cancer in women. Moreover, CRC-related deaths accounted for 9.3% of cancer-related deaths, making it the second leading cause of cancer mortality after lung cancer (1–4).

Nowadays, chronic kidney disease (CKD) has become a global public health problem, and Asia has one of the highest prevalence of CKD (5–7). According to statistics, in 2019, there were 9.8 million new CKD cases and 763,024 CKD-related deaths in Asia (8, 9).

Several studies have shown that CKD was significantly associated with an increased incidence of CRC (10–12). However, the precise effect of CKD on postoperative complications and prognosis in CRC remains a subject of debate. While Currie A et al. suggested that CKD patients might be more likely to develop cardiovascular complications after CRC resection and had an increased risk of disease-free survival (DFS) (13); Huang CS et al. found that the CKD group had a significantly lower 3-year DFS rate compared to the Non-CKD group (14); Moreover, Nozawa H et al. concluded that CKD had little effect on overall survival (OS) in TNM stage III CRC patients (15).

As previous studies reported, it was unclear the effect of CKD on postoperative complications and prognosis in CRC, Therefore, the purpose of the current study was to analyze the effect of CKD on the short-term outcomes and prognosis of CRC patients undergoing primary surgery for CRC.

## Methods

### Patients

CRC patients who underwent radical surgery were included from Jan 2011 to Jan 2020 in a single clinical teaching hospital. This study was performed following the World Medical Association Declaration of Helsinki. And this study obtained Ethical approval from the institutional review board (The First Affiliated Hospital of Chongqing Medical University, 2024–010-01). All patients participating in the study obtained written informed consent.

### Inclusion and exclusion criteria

**Figure 1:**
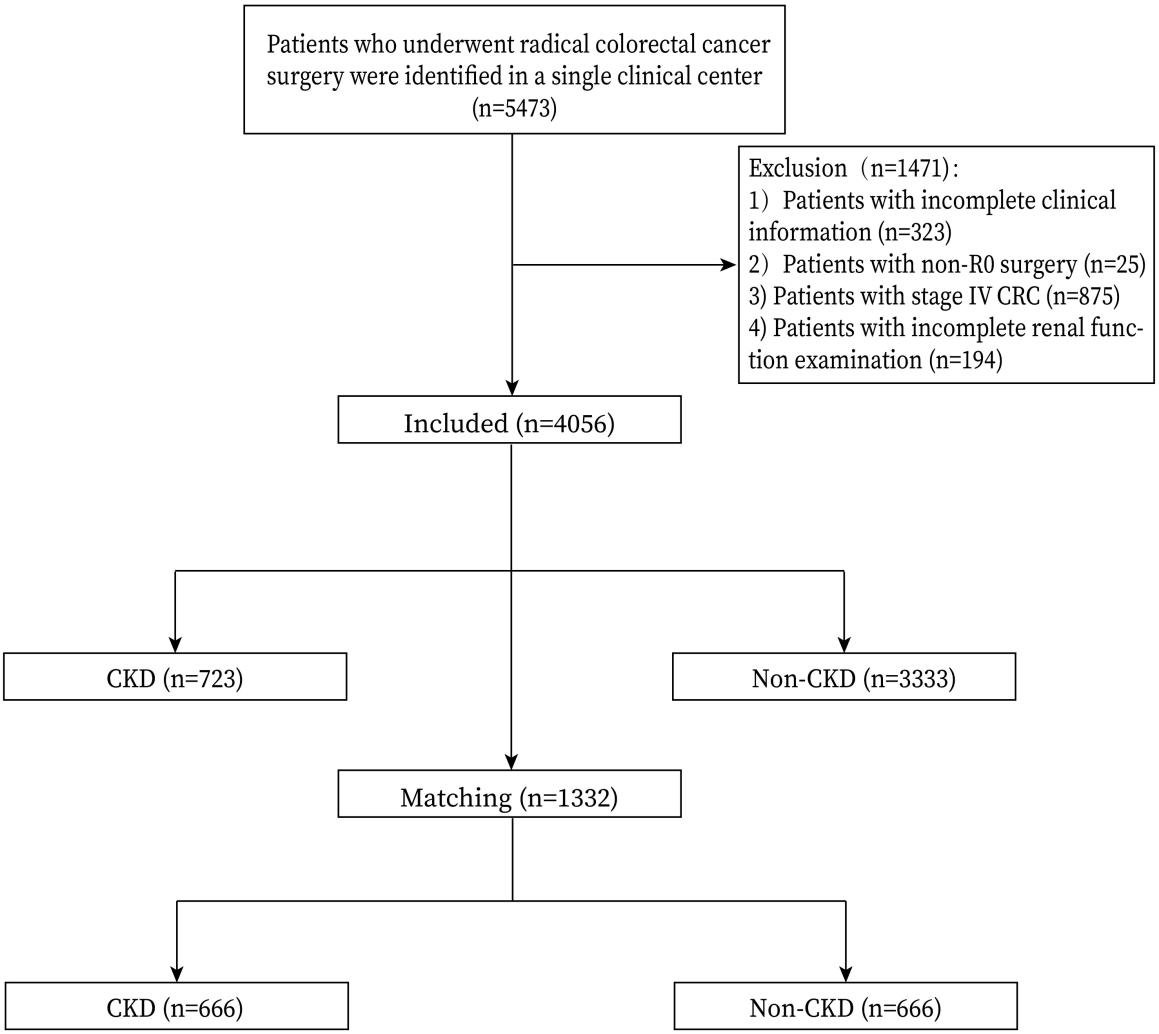
Flow chart of patient selection.

Patients who underwent primary CRC surgery were included in this study (n=5473). The exclusion criteria were as follows: 1, patients with incomplete clinical information (n=323); 2, non-R0 surgery (n=25); 3, stage IV CRC (n=875); and 4, incomplete renal function examination (n=194). Finally, a total of 4056 patients were included in this study. The flowchart and inclusion and exclusion criteria were shown in **Figure 1**.

### Surgery management and follow-up

All included patients underwent total mesorectal resection or total mesocolic resection based on the oncological principles, with postoperative pathology confirming R0 resection. Routine follow-up was performed after surgery: every three months for three years, and every six months thereafter.

### Definitions

We used the Chronic Kidney Disease Epidemiology Collaboration (CKD-EPI) creatinine equation to calculate eGFR to assess renal function in all the included patients. The CKD-EPI equation was gender-specific, and the specific formula was as follows: eGFR = 141 × min (Scr/κ, 1)α × max (Scr/κ, 1)- 1.209× 0.993 Age× 1.018 [if female], where Scr was serum creatinine, κ was 0.7 for females and 0.9 for males, α was −0.329 for females and −0.411 for males, min indicated the minimum of Scr/κ or 1, and max indicated the maximum of Scr/κ or 1 (16, 17). CKD was defined as persistent proteinuria or eGFR<60ml/min/1.73m^2^ (18), therefore, we defined patients with eGFR< 60ml/min/1.73m^2^ as the CKD group and those with eGFR≥ 60ml/min/1.73m^2^ as the Non-CKD group. The pathological staging of tumors was basically based on the AJCC 8th edition diagnosis (19). Complications were defined according to the Clavien- Dindo classification (20) and major complications were defined as Clavien- Dindo classification ≥ III complications. III Complications Includes complications requiring surgical, endoscopic, or radiologic intervention; OS was defined as the time from the date of surgery to the individual patients’ death of any cause or the last follow-up time; DFS was defined as the time from the date of surgery to the date of radiographic or pathological confirmation of recurrence, death or the last follow-up.

### Data collection

We retrospectively collected baseline information and postoperative short-term outcomes through the inpatient system. Baseline information included age, gender, body mass index (BMI), smoking and drinking history, hypertension, type 2 diabetes mellitus (T2DM), tumor location, tumor size and tumor stage (**Table 1**). Postoperative short-term outcomes included operative time, blood loss, hospital stay, overall complications and major complications (**Table 2**). Survival data were mainly obtained through the outpatient follow-up system and telephone interviews.

**Table 1:** Baseline characteristics of CKD before and after PSM.

Characteristics	Before PSM			After PSM		
	CKD (723)	Non-CKD (3333)	P value	CKD (666)	Non-CKD (666)	P value
eGFR, ml/min/1.73 m^2^	47.9 ± 11.4	85.3 ± 17.5	<0.01**	48.2 ± 11.3	76.9 ± 17.7	<0.01**
Age (year)	70.6 ± 10.2	61.2 ± 11.9	<0.01**	69.7 ± 10.0	69.5 ± 9.1	0.718
Sex			<0.01**			0.506
Male	609 (84.2%)	1780 (53.4%)		552 (82.9%)	561 (84.2%)	
Female	114 (15.8%)	1553 (46.6%)		114 (17.1%)	105 (15.8%)	
BMI (kg/m^2^)	23.0 ± 3.2	22.6 ± 3.2	0.013*	22.9 ± 3.2	23.0 ± 3.2	0.751
Smoking	368 (50.9%)	1166 (35.0%)	<0.01**	339 (50.9%)	340 (51.1%)	0.956
Drinking	269 (37.2%)	972 (29.2%)	<0.01**	255 (38.3%)	251 (37.7%)	0.821
Hypertension	317 (43.8%)	747 (22.4%)	<0.01**	273 (40.1%)	257 (38.6%)	0.370
T2DM	119 (16.5%)	380 (11.4%)	<0.01**	106 (15.9%)	102 (15.3%)	0.763
Tumor location			0.238			0.411
Colon	355 (49.1%)	1556 (46.7%)		326 (48.9%)	311 (46.7%)	
Rectum	368 (50.9%)	1777 (53.3%)		340 (51.1%)	355 (53.3%)	
Tumor size			0.882			0.616
< 5cm	423 (58.5%)	1940 (58.2%)		391 (58.7%)	400 (60.1%)	
≥ 5cm	300 (41.5%)	1393 (41.8%)		275 (41.3%)	266 (39.9%)	
Tumor stage			0.817			0.510
I	146 (20.2%)	650 (19.5%)		130 (19.6%)	117 (17.6%)	
II	314 (43.4%)	1431 (42.9%)		298 (44.7%)	317 (47.6%)	
III	263 (36.4%)	1252 (37.6%)		238 (35.7%)	232 (34.8%)	

Variables are expressed as the mean ± SD, n (%), *P-value <0.05, ** P-value <0.01.

CKD, chronic kidney disease; eGFR, estimated glomerular filtration rate; T2DM, type 2 diabetes mellitus; BMI, body mass index; PSM, propensity score matching.

**Table 2:** Short-term outcomes before and after PSM.

Characteristics	Before PSM			After PSM		
	CKD (723)	Non-CKD (3333)	P value	CKD (666)	Non-CKD (666)	P value
Operation time (min)	222.8 ± 83.4	225.6 ± 80.2	0.400	222.5 ± 84.8	226.6 ± 84.9	0.376
Blood loss (mL)	107.7 ± 148.0	95.6 ± 127.7	0.025*	106.6 ± 148.5	101.2 ± 136.4	0.498
Hospital stay (day)	12.9 ± 13.2	10.8 ± 7.3	<0.01**	12.7 ± 12.9	11.2 ± 7.1	0.009**
Overall complications	219 (30.3%)	669 (20.1%)	<0.01**	199 (29.9%)	167 (25.1%)	0.050*
Major complications	28 (3.9%)	67 (2.0%)	0.003**	25 (3.8%)	14 (2.1%)	0.358

Variables are expressed as the mean ± SD, n (%), *P-value <0.05, **P-value <0.01.

Abbreviations: CKD, chronic kidney disease; PSM, propensity score matching.

### Propensity score matching (PSM)

Since the grouping of the CKD group and the Non-CKD group was non-random, and variables were unbalanced. Therefore, we used PSM in this study. Propensity scores are most commonly estimated by logistic regression, where treatment group is considered the outcome and regressed against observed baseline characteristics (21). We initiated a 1:1 (CKD group vs Non-CKD group) matching analysis by PSM and nearest neighbor matching algorithms and specified a caliper width with 0.01 standard deviation to adjust for differences in baseline characteristics between the two groups. Baseline information for PSM included age, gender, BMI, smoking and drinking history, hypertension, T2DM, tumor location, tumor size and tumor stage.

### Statistical analysis

In this article, continuous variables were expressed as mean ± SD, and Frequency variables were expressed as n (%). We use independent samples t-test to analyze differences in eGFR, age, BMI, operation time, blood loss and hospital stay between the CKD group and the Non-CKD group. The chi-square test or Fisher’s exact test was used to analyze differences in sex, smoking, drinking, hypertension, T2DM, tumor location, tumor size, tumor stage, overall complications and major complication between the CKD group and the Non-CKD group. Furthermore, to identify independent predictors of OS and DFS, we used the Cox regression analysis. Firstly, we performed univariate analysis for CKD, Sex, BMI, T2DM, Tumor site, Tumor stage, Smokin, Drinking, Hypertension, Tumor size, Overall complications in the two groups, and then multivariate analysis for factors with P-value <0.05 after univariate analysis. Data were analyzed using the SPSS (version 26.0) statistical software, and a bilateral p-value < 0.05 was considered statistically significant.

## Results

### Patients

According to the relevant inclusion and exclusion criteria, a total of 4056 patients undergoing CRC surgery were finally included in this analysis, including 723 patients in the CKD group and 3333 patients in the Non-CKD group. After 1:1 PSM, there were 666 patients in each group (**Figure 1**)

### Baseline characteristics

Before PSM, there were significant differences in the baseline information between the CKD group and the Non-CKD group. The CKD group had older age (P<0.01), a higher proportion of male (P<0.01), higher BMI (P=0.013), higher proportions of smoking and drinking (P<0.01), and more patients with hypertension and T2DM (P<0.01). After 1:1 PSM, there was no significant difference in baseline information between the two groups (p>0.05) (**Table 1**)

### Short-term outcomes

Compared the short-term postoperative outcomes between the CKD group and the Non-CKD group, we found that the CKD group had more blood loss (P=0.025), longer postoperative hospital stay (P<0.01), more overall complications (P<0.01) and more major complications (P=0.003). After PSM, the CKD group still had a longer postoperative hospital stay (P=0.009) and a higher incidence of overall complications (p=0.050). CKD might not affect major complications (P=0.358) (**Table 2**).

### The incidence of the postoperative complications

This study counted the postoperative complications with higher morbidity, such as intestinal obstruction, lymphatic fistula, anastomotic fistula, thrombus, postoperative death and pneumonia. Before PSM, the incidence of intestinal obstruction, lymphatic fistula, anastomotic fistula, thrombus, postoperative death and pneumonia in the CKD group was 2.4%, 0.3%, 2.5%, 1.9%, 0.7%, and 6.4%, respectively. The incidence of intestinal obstruction, lymphatic fistula, anastomotic fistula, thrombus, postoperative death and pneumonia in the Non-CKD group was 1.8%, 0.5%, 2.3%, 0.8%, 0.2%, and 2.3%, respectively. After PSM, the incidence of intestinal obstruction, lymphatic fistula, anastomotic fistula, thrombus, postoperative death and pneumonia in the CKD group was 2.6%, 0.3%, 2.6%, 2.0%, 0.7%, 5.6%, respectively. The incidence of intestinal obstruction, lymphatic fistula, anastomotic fistula, thrombus, postoperative death and pneumonia in the Non-CKD group was 2.9%, 0.7%, 3.6%, 0.7%, 0.2%, 4.8%, respectively.

### Univariate and multivariate analysis of OS and DFS before and after PSM

The median follow-up time was 33 (1–114) months. Before PSM, in terms of OS, age (p<0.01, HR=1.039, 95% CI=1.031–1.048), tumor stage (p<0.01, HR=2.094, 95% CI=1.827–2.401, tumor size (p=0.005, HR=1.275, 95% CI=1.075–1.511) and overall complications (p<0.01, HR=1.664, 95% CI=1.391–1.990) were independent predictors. Regarding DFS, age (p<0.01, HR=1.028, 95% CI=1.020–1.035), tumor stage (p<0.01, HR=2.022, 95% CI=1.792–2.281), and overall complications (p<0.01, HR=1.532, 95% CI=1.300–1.805) were independent predictors. However, CKD was not an independent predictor of OS or DFS (OS: p=0.440, HR=1.085, 95% CI=0.882–1.335); DFS: p=0.122, HR=1.160, 95% CI=0.961–1.399) (**Table 3**).

**Table 3:** Univariate and multivariate analysis of overall survival of the whole cohort and disease free survival of the whole cohort before PSM.

Risk factors	Univariate analysis		Multivariate analysis	
	HR (95% CI)	P value	HR (95% CI)	P value
OS				
CKD (yes/no)	1.573 (1.296-1.909)	<0.01**	1.085 (0.882-1.335)	0.440
Age (years)	1.045 (1.037-1.053)	<0.01**	1.039 (1.031-1.048)	<0.01**
Sex (male/female)	0.877 (0.737-1.044)	0.141		
BMI (kg/m^2^)	0.952 (0.926-0.978)	<0.01**	0.979 (0.953-1.006)	0.133
T2DM (yes/no)	1.265 (0.990-1.618)	0.060		
Tumor site (colon/rectum)	1.162 (0.980-1.377)	0.084		
Tumor stage (III/II/I)	2.116 (1.850-2.420)	<0.01**	2.094 (1.827-2.401)	<0.01**
Smoking (yes/no)	1.045 (0.878-1.244)	0.621		
Drinking (yes/no)	1.006 (0.836-1.210)	0.954		
Hypertension (yes/no)	0.998 (0.821-1.213)	0.984		
Tumor size (≥ 5cm/<5cm)	1.451 (1.224-1.720)	<0.01**	1.275 (1.075-1.511)	0.005**
Overall complications (yes/no)	1.872 (1.567-2.235)	<0.01**	1.664 (1.391-1.990)	<0.01**
DFS				
CKD (yes/no)	1.522 (1.276-1.814)	<0.01**	1.160 (0.961-1.399)	0.122
Age (years)	1.033 (1.026-1.040)	<0.01**	1.028 (1.020-1.035)	<0.01**
Sex (male/female)	0.886 (0.758-1.036)	0.130		
BMI (kg/m^2^)	0.973 (0.950-0.997)	0.028*	0.993 (0.969-1.017)	0.566
T2DM (yes/no)	1.116 (0.888-1.403)	0.347		
Tumor site (colon/rectum)	1.081 (0.928-1.260)	0.315		
Tumor stage (III/II/I)	2.037 (1.808-2.295)	<0.01**	2.022 (1.792-2.281)	<0.01**
Smoking (yes/no)	1.068 (0.914-1.249)	0.407		
Drinking (yes/no)	1.018 (0.862-1.201)	0.835		
Hypertension (yes/no)	1.009 (0.847-1.201)	0.922		
Tumor size (≥ 5cm/<5cm)	1.306 (1.121-1.521)	<0.01**	1.160 (0.995-1.352)	0.057
Overall complications (yes/no)	1.683 (1.430-1.980)	<0.01**	1.532 (1.300-1.805)	<0.01**

*P-value <0.05, ** P-value <0.01.

HR, hazard ratio; CI, confidence interval; BMI, body mass index; T2DM, type 2 diabetes mellitus; CKD, chronic kidney disease.

After PSM, in terms of OS, age (p<0.01, HR=1.045, 95% CI=1.028–1.062), tumor stage (p<0.01, HR=1.931, 95% CI=1.564–2.385) and overall complications (p<0.01, HR=1.858, 95% CI=1.423–2.425) were independent predictors. Regarding DFS, age (p<0.01, HR=1.034, 95% CI=1.020–1.049), tumor stage (p<0.01, HR=1.852, 95% CI=1.537–2.231), and overall complications (p<0.01, HR=1.651, 95% CI=1.295–2.10) were independent predictors. However, CKD was not an independent predictor of OS or DFS (OS: p=0.619, HR=1.070, 95% CI=0.820–1.396; DFS: p=0.472, HR=1.092, 95% CI=0.859–1.389) (**Table 4**).

**Table 4:** Univariate and multivariate analysis of overall survival of matching cohort and disease free survival of matching cohort after PSM.

Risk factors	Univariate analysis		Multivariate analysis	
	HR (95% CI)	P value	HR (95% CI)	P value
OS				
CKD (yes/no)	1.070 (0.820-1.396)	0.619		
Age (years)	1.054 (1.038-1.070)	<0.01**	1.045 (1.028-1.062)	<0.01**
Sex (male/female)	1.391 (1.010-1.915)	0.043*	1.106 (0.791-1.547)	0.554
BMI (kg/m^2^)	0.955 (0.916-0.996)	0.032*	0.983 (0.941-1.026)	0.433
T2DM (yes/no)	1.227 (0.868-1.734)	0.246		
Tumor site (colon/rectum)	1.422 (1.091-1.853)	0.009*	1.223 (0.936-1.599)	0.141
Tumor stage (III/II/I)	1.987 (1.614-2.447)	<0.01**	1.931 (1.564-2.385)	<0.01**
Smoking (yes/no)	0.886 (0.680-1.153)	0.368		
Drinking (yes/no)	0.838 (0.633-1.109)	0.217		
Hypertension (yes/no)	0.793 (0.601-1.047)	0.102		
Tumor size (≥ 5cm/<5cm)	1.240 (0.952-1.615)	0.110		
Overall complications (yes/no)	1.986 (1.523-2.590)	<0.01**	1.858 (1.423-2.425)	<0.01**
DFS				
CKD (yes/no)	1.092 (0.859-1.389)	0.472		
Age (years)	1.041 (1.027-1.055)	<0.01**	1.034 (1.020-1.049)	<0.01**
Sex (male/female)	1.348 (1.007-1.806)	0.045*	1.144 (0.846-1.556)	0.383
BMI (kg/m^2^)	0.979 (0.943-1.017)	0.270		
T2DM (yes/no)	1.121 (0.814-1.544)	0.485		
Tumor site (colon/rectum)	1.253 (0.987-1.591)	0.063		
Tumor stage (III/II/I)	1.987 (1.614-2.447)	<0.01**	1.852 (1.537-2.231)	<0.01**
Smoking (yes/no)	0.894 (0.704-1.134)	0.355		
Drinking (yes/no)	0.876 (0.681-1.126)	0.300		
Hypertension (yes/no)	0.885 (0.691-1.133)	0.331		
Tumor size (≥ 5cm/<5cm)	1.166 (0.918-1.482)	0.207		
Overall complications (yes/no)	1.750 (1.373-2.229)	<0.01**	1.651 (1.295-2.10)	<0.01**

*P-value <0.05, ** P-value <0.01.

HR, hazard ratio; CI, confidence interval; BMI, body mass index; T2DM, type 2 diabetes mellitus; CKD, chronic kidney disease.

## Discussion

A total of 4056 patients with CRC surgery were included in this analysis finally. After PSM, there were 666 in the CKD group and the Non-CKD group, respectively. After PSM, the CKD group had a longer postoperative hospital stay. However, CKD was not an independent predictor of OS or DFS.

The effect of CKD on the surgical outcomes has been a hot topic. Aune D et al. believed that after cardiac surgery, patients with end-stage renal disease had a significantly higher mortality rate than patients with normal renal function (6) Ciriaco P et al. concluded that hemodialysis (HD) patients who underwent pneumonectomy had a higher incidence of postoperative complications (22); Han IH et al. concluded that patients with end-stage renal disease who underwent spinal surgery had higher morbidity and mortality (23). We concluded that CKD was associated with an increased risk of most surgical outcomes, but the effect of CKD on patients after CRC surgery was controversial. Some studies suggested that CKD increased postoperative morbidity and mortality in CRC patients (12, 13, 24–27). Therefore, the purpose of this study was to analyze the effect of CKD on short-term outcomes and prognosis of CRC undergoing primary surgery.

Our study showed that CKD patients who underwent CRC surgery had longer hospital stays after harmonizing the differences in baseline data, which was consistent with previous studies (12, 13). Many clinicians believed that the kidneys and the heart were two organs that interacted with each other (28–31). Studies had shown that CKD was associated with a significantly increased risk of myocardial infarction, coronary artery disease, left ventricular hypertrophy and death from cardiac causes (28–32). In addition, CKD led to elevated levels of inflammatory factors, arterial hypercalcification and endothelial dysfunction (32). All of these mechanisms might contribute to delayed postoperative recovery after surgery, thereby increasing the length of hospital stay. Additionally, this endothelial injury could explain the pro-inflammatory environment in CKD due to uremia, malnutrition, volume overload, or altered calcium and phosphorus metabolism (32). Since inflammatory mediators might lead to malignancy through induction of precancerous mutations, adaptive responses, and environmental changes (33), this also confirmed the association of CKD with the incidence of CRC (7–9).

In this present study, age, tumor stage, and overall complications were independent predictors of OS and DFS, consistent with previous studies (34, 35). Therefore, we should pay attention to the control of operative complications. However, it was worth noting that in the PSM analysis of major postoperative complications, OS and DFS in CRC patients, no significant difference was found between the CKD group and the Non-CKD group. Even though the CKD group showed a higher overall postoperative complication rate before and after PSM, the incidence of major complications was similar between groups. Despite this higher incidence of overall complications, the OS and DFS remained similar between CKD and Non-CKD groups after PSM. Therefore, further research is warranted to elucidate the precise impact of CKD on prognosis.

After reviewing the relevant studies, we learned that although there were few previous studies on the effect of CKD on CRC surgery, this study was the first to use PSM to analyze the impact of CKD on the short-term outcomes and prognosis of CRC patients. The use of PSM greatly reduced differences in the baseline characteristics and made conclusions more reliable. And compared to conventional PSM articles, this study also analyzed pre-matching data, which better illustrated the effect size of potential confounding factors. However, this study also had some limitations. First, this was a retrospective single-center study; second, the follow-up time was relatively short; third, this study only compared the CKD group and the Non-CKD group, and lacked specific staging studies of CKD; fourth, our results might still be biased due to selection bias stemming from unbalanced data. Therefore, we are looking forward to more comprehensive multicenter prospective randomized controlled studies to further confirm our findings in the future. In future research, we also would like to use advanced methodologies, such as Cox regression with shared frailty, to enhance the methodological robustness and better align with the standards of academic excellence.

In conclusion, CKD prolonged postoperative hospital stay; however, CKD might not affect major postoperative complications, OS or DFS of CRC.

## Data availability statement

The raw data supporting the conclusions of this article will be made available by the authors, without undue reservation.

## Ethics statement

The studies involving humans were approved by the First Affiliated Hospital of Chongqing Medical University. The studies were conducted in accordance with the local legislation and institutional requirements. The participants provided their written informed consent to participate in this study. Written informed consent was obtained from the individual(s) for the publication of any potentially identifiable images or data included in this article.

## Author contributions

C-YW: Writing – review & editing. S-PQ: Writing – original draft. S-QR: Writing – review & editing. Z-XH: Writing – review & editing.
